# Anxiety Level of University Students During COVID-19 in Saudi Arabia

**DOI:** 10.3389/fpsyt.2020.579750

**Published:** 2020-12-11

**Authors:** Heba Bakr Khoshaim, Areej Al-Sukayt, Karuthan Chinna, Mohammad Nurunnabi, Sheela Sundarasen, Kamilah Kamaludin, Gul Mohammad Baloch, Syed Far Abid Hossain

**Affiliations:** ^1^Deanship of Educational Services, Prince Sultan University, Riyadh, Saudi Arabia; ^2^Department of Accounting, Prince Sultan University, Riyadh, Saudi Arabia; ^3^Faculty of Health and Medical Sciences, School of Medicine, Taylor's University, Subang Jaya, Malaysia; ^4^College of Business Administration, IUBAT - International University of Business Agriculture and Technology, Dhaka, Bangladesh

**Keywords:** pandemic, university students, anxiety, Saudi Arabia, COVID-19, women anxiety

## Abstract

COVID-19 is the worst pandemic of this millennium, and it is considered to be the “public enemy number one.” This catastrophe has changed the way we live in the blink of an eye. Not only has it threatened our existence and health status, but the damage associated with it could equally affect our economic, social, and educational systems. The focus of this study was on the anxiety level of university students during the COVID-19 pandemic in Saudi Arabia. The study was conducted between March and June 2020. A questionnaire was administered online, and 400 completed questionnaires were returned. In this study, the Zung self-rating anxiety scale was used to determine the anxiety levels among the respondents. The results indicated that about 35% of the students experienced moderate to extreme levels of anxiety. Anxiety was highly associated with age, sex, and level of education. These findings can enlighten government agencies and policy makers on the importance of making prompt, effective decisions to address students' anxiety during the COVID-19 pandemic. Researchers are encouraged to focus their future studies on how to develop strategies to boost students' resilience and enhance their adaptability skills for similar disasters in the future.

## Introduction

The COVID-19 disease is the worst pandemic outbreak in the new millennium. Caused by the *severe acute respiratory syndrome coronavirus 2* (SARS-CoV-2), the first case was detected in December 2019 in Wuhan, China. Since then, the disease has spread to almost every part of the globe. The spread of the disease was so fast that on January 30, 2020, the WHO declared COVID-19 to be a *Public Health Emergency of International Concern*. As of April 2020, almost 3 million positive cases were confirmed worldwide, with about 200,000 fatalities (1). In view of the high number of secondary cases arising from one primary case and the population being largely susceptible to infection, the WHO declared COVID-19 disease as a pandemic on March 12, 2020 (2). To control the spread of the disease, China and many other countries imposed lockdowns, either nationwide or in places severely affected by the virus. Educational institutions, financial institutions, centers of economic activities, and amusement centers closed indefinitely (2, 3). It is estimated that as of April 2020, more than 300 million students were affected by COVID-19 globally (4). Worldwide, many schools and colleges either closed or resorted to distance learning. Public gathering and celebrations were prohibited. People with severe infection were treated in hospitals, and the less severe patients were placed in quarantine centers. These measures are similar to those that have previously proven effective during the H1N1 pandemic as reported by Sakaguchi et al. (5) and during SARS as reported by Ries (6).

In the Kingdom of Saudi Arabia (KSA), the Ministry of Health (MOH) announced the first COVID-19 case on March 2, 2020, and by the end of the month, 154 new COVID-19 cases were reported (7). Anticipating widespread infection, government agencies swiftly implemented several control measures to combat the spread of the virus. In view of mass gatherings, Umrah in Mecca and visitations to the Prophet's Mosque in Madina were suspended immediately, and all the mosques in the country closed temporarily (8–10). Quarantining of infected people and practicing social distancing became the norm. Universities and schools were closed, switching to virtual classes, to ensure uninterrupted teaching. Students were expected to continue studies from their place of residence. Despite the awareness campaigns and precautionary measures taken by the government, the number of cases continued to increase. Within 3 months, the confirmed cases escalated to 98,869, out of which 71,791 recovered, and 642 died (11).

In addition to the risk of infection and possible death, an epidemic also exerts tremendous psychological pressure on people worldwide (12–17). Several studies have discussed the short- and long-term effects of epidemics on the social and psychological well-being in the population (18–20). Those who were tested positive for a disease continue to be stigmatized and suffer from seclusion in their own society even after they have recovered (19, 21). Those quarantined experienced psychological stressors including “longer quarantine duration, infection fears, frustration, limited supplies, insufficient communication, financial loss, and stigma” [21, p. 1]. Brooks et al. (22) expect that COVID-19 will result in drastic post-traumatic stress symptoms, confusion, and anger.

Moreover, anxiety level among college-level students is already a public health concern. In fact, several previous researches have examined students' anxiety, depression, and stress and have discussed factors that might affect students' mental health (23–32). Looking at students in the Kingdom of Saudi Arabia, Amr et al. (27) reported a 20% level of anxiety among college-level Saudi students, while Al-Gelban (26) argued that 14.3% of secondary school students experience anxiety. In contrast, Al-Gelban (25) and Al-Gelban et al. (33) reported a much higher anxiety level (48.9 and 66.2%, respectively) among high school students. Given that the spread of COVID-19 and the switch to virtual learning are unprecedented and unexpected experiences in Saudi Arabia, we can expect such circumstances to be associated with major psychological challenges for the students. The fear of getting infected or losing loved ones to the disease while having to rapidly adjust to the new teaching and assessment procedures would have had a tremendous pressure on the students. At this time, besides testing, planning, and implementing new teaching and learning environments, educational institutions need to assess the students' psychological well-being so that appropriate measures can be taken to help students cope with unprecedented changes. The purpose of this study is to assess the anxiety status of students in Saudi Arabia during COVID-19 pandemic.

## Research Methodology

### Participants, Procedures, and Timeline of Survey

This study examined the anxiety level among university students in Saudi Arabia, specifically in Riyadh, at the time of the COVID-19 pandemic. Riyadh is the capital of Saudi Arabia and one of the largest cities in the Kingdom, with an area of 1,798 km^2^ and a population estimated at 7,231,447 (34). The sample was chosen from one private university. The total number of undergraduate students of both genders currently enrolled in this university is 5,057 students, comprising 3,085 female and 1,972 male students. An online questionnaire created in Google Forms was distributed via email to all undergraduate students at this university. Data were collected from April 20, 2020, to June 6, 2020. Although this period is not ideal as it is at the end of the academic year, the authors preferred to address this concern while the COVID-19 concern is at its maximum instead of waiting until the end of summer. The students were briefed on the purpose, and anonymity and confidentiality of their responses. Permission to conduct this study was obtained from the Institutional Review Board (IRB) of the university.

### Research Instrument

In this study, the anxiety level was assessed using Zung's self-rating anxiety questionnaire, a validated 20-item self-report instrument (35) with reported Cronbach's alpha = 0.897 and internal correlation = 0.913 (36). The instrument employs a four-point Likert scale where: “1 = Never or very rare,” “2 = Sometimes,” “3 = Often,” and “4 = Very Often or always.” Questions 1–5 characterize the emotional pointers of anxiety, whereas questions 6–20 signify the physical symptoms of anxiety. For each respondent, the sum of the scores for 20 items ranges from 20 to 80. The sum of scores are then converted to an “Anxiety Index” with values ranging from 25 to 100. Following the recommendations from Zung (37) and Dunstan and Scott (38), an Anxiety Index <45 indicates “Anxiety within normal range,” a value in the range of 45–59 indicates “Mild to moderate anxiety,” a value in the range of 60–74 indicates “Marked to severe anxiety,” and values ≥75 indicates “Most extreme anxiety.” Apart from Zung's self-rating anxiety scale, demographic information such as age, gender, year of study, field of study, and living arrangements during the pandemic were also recorded. Moreover, the participants had the chance to reflect on their feelings through open-ended sections. Although Arabic is the mother tongue of the Saudi population, English is the language of instruction at this university. Hence, the instrument was used in its original language and was not translated.

### Data Analysis

The IBM SPSS version 22 software (39) was used in data analysis. Chi-square and ordinal regression procedures were used to determine the factors associated with levels of anxiety. All the variables that were significant at 0.25 level (40) in the chi-square tests were tested in ordinal logistic regression analysis.

### Ethical Clearance

Permission to conduct this study was obtained from the Institutional Review Board (IRB) of the university.

## Results

The aim of this study was to examine the anxiety level of university students in Saudi Arabia during the COVID-19 pandemic.

### Demographic Characteristics

The questionnaire was sent to 5,057 undergraduate students, which comprise the population of this university at that time. A total of 400 responses were received, which represents an 8% response rate. Although this is a low response rate, it might be because the questionnaire was sent at the end of the semester and the beginning of final exams. The demographic characteristics of the respondents are shown in Table 1. Out of the 400 respondents, 75.25% (301) were females, and 24.75% (99) were males and most of the respondents (93.5%). The skewed response rate toward females could be due to the fact that the females represent around 60% (3,085 female students) of the population of the university. Most of the respondents (93.5%) were in the age group of 19–25 years. Regarding their field of study, about one-third of the students were from the College of Business Administration, and around one-sixth each were from the College of Law and the College of Computer and Information Sciences. Most of the students (80%) were undergraduates, and 20% were in the Preparatory Year Program (PYP), which is a compulsory 1-year program for all high school graduates. In terms of accommodation, 89.8% (359) were living at homes owned by their parents, and only around 10% were living at rented facilities. Moreover, 94.2% of the students were staying with their families at the time of the pandemic.

**Table 1:** Demographic characteristics of the respondents.

**Variable**	**Frequency**	**Percentage**
**Gender**		
Female	301	75.2%
Male	99	24.8%
**Age**		
≤18 years	15	3.8%
19–25 years	374	93.5%
≥26	11	2.8%
**College**		
Business Administration	131	32.8%
Computing and IS	73	18.2%
Engineering	65	16.2%
Humanities	16	4.0%
Law	69	17.2%
Preparatory year program	46	11.5%
**Level of study**		
Preparatory year program	79	19.8%
Undergraduate	321	80.2%
**Year of study**		
Year 1	127	31.8%
Year 2	72	18.0%
Year 3	74	18.5%
Year 4	72	18.0%
Year 5 and above	55	13.8%
**Current accommodation**		
Family home	359	89.8%
Rented premises	41	10.2%
**Currently staying with**		
Family/relatives	377	94.2%
Alone	23	5.8%

### Levels of Anxiety

Among the respondents, 21.5% (86), 8.8% (35), and 4.3% (17) experienced “minimal to moderate,” “marked to severe,” and “most extreme” levels of anxiety, respectively. For further analysis, respondents in the “marked to severe” and “most extreme” anxiety category were grouped together as “severe to extreme” level of anxiety. A summary of the results is shown in Table 2.

**Table 2:** Anxiety levels based on Zung's classification.

**Anxiety**	**Frequency**	**Percentage**	**Anxiety**	**Frequency**	**Percentage**
Normal	262	65.5%	Normal	262	65.5%
Minimal to moderate	86	21.5%	Minimal to moderate	86	21.5%
Marked to severe	35	8.8%	Severe to extreme	52	13.0%
Most extreme	17	4.3%			

### Factors Associated With College Students' Anxiety During the Epidemic

#### Results From Univariate Analysis

In the univariate analyses, chi-square tests were used to determine the associations between students'demographic variables and anxiety levels. The results are shown in Table 3. Among the demographic variables, gender, age, year of study, and living arrangement were significant at a 0.25 level.

**Table 3:** Results of univariate analyses.

**Variable**	**Normal**	**Minimal to moderate**	**Severe to extreme**	**Chi-square**	***p*-value**
**Gender**				7.465	0.024
Female	186 (61.8%)	71 (23.6%)	44 (14.6%)		
Male	76 (76.8%)	15 (15.2%)	8 (8.1%)		
**Age**				3.277	0.194
≤18	7 (46.7%)	4 (26.7%)	4 (26.7%)		
≥19	255 (96.2%)	82 (21.3%)	48 (12.5%)		
**College**				6.862	0.738
Business administration	90 (68.7%)	28 (21.4%)	13 (9.9%)		
Computing and IS	49 (67.1%)	15 (20.5%)	2 (12.3%)		
Engineering	38 (58.5%)	17 (26.2%)	10 (15.4%)		
Humanities	10 (62.5%)	5 (31.2%)	1 (6.2%)		
Law	43 (62.3%)	15 (21.7%)	11 (15.9%)		
Preparatory year program	32 (69.6%)	6 (13.0%)	8 (17.4%)		
**Level of study**				0.918	0.632
PYP	55 (69.6%)	14 (17.7%)	10 (12.7%)		
Undergraduate	207 (64.5%)	72 (22.4%)	42 (13.1%)		
**Year of study**				12.569	0.129
Year 1	86 (67.7%)	22 (17.3%)	19 (15.0%)		
Year 2	50 (69.4%)	17 (23.6%)	5 (6.9%)		
Year 3	48 (64.9%)	16 (21.6%)	10 (13.5%)		
Year 4	37 (51.4%)	23 (31.9%)	12 (16.7%)		
Year 5 and above	41 (74.5%)	6 (14.5%)	6 (10.9%)		
**Accommodation**				0.630	0.730
Family home	233 (64.9%)	79 (22.0%)	47 (13.1%)		
Rented premises	29 (70.7%)	7 (17.1%)	5 (12.2%)		
**Living arrangement**				3.488	0.175
Alone	14 (60.9%)	8 (34.8%)	1 (4.3%)		
Family/Relatives	248 (645.8%)	78 (50.7%)	51 (13.5%)		

#### Results From Ordinal Regression Analysis

The variables of gender, age, year of study, and living arrangement that were significant at the 0.25 level in the univariate analyses were further tested using ordinal logistic regression analysis. In this analysis, only gender and year of study were significant (Table 4). Interestingly, female students were more prone to higher levels of anxiety compared to males (OR = 1.963, 95% CI = 1.160, 3.322, *P* = 0.012). Students in their fourth year were more anxious compared to students in their fifth year or final year (OR = 2.440, 95% CI = 1.150, 5.179, *P* = 0.020).

**Table 4:** Results from ordinal multivariate analysis.

**Parameter**	**B**	**SE**	***p*-value**	**OR_**adj**_ (95% CI)**
**Gender**				
Female	0.674	0.269	0.012	1.963 (1.160, 3.322)
Male	*ref*			1
**Age**				
≤18 years	0.870	0.530	0.101	2.386 (0.845, 6.739)
≥19 years	*ref*			1
**Year of study**				
Year 1	0.155	0.3723	0.678	1.167 (0.563, 2.422)
Year 2	0.139	0.4020	0.729	1.149 (0.523, 2.537)
Year 3	0.394	0.3927	0.316	1.483 (0.687, 3.201)
Year 4	0.892	0.3840	0.020	2.440 (1.150, 5.179)
Year 5 and above	*ref*			1
**Living arrangement**				
Alone	−0.116	0.431	0.780	0.891 (0.382, 2.074)
Family/Relatives	*ref*			1

*B, regression coefficient; SE, standard error; OR, odds ratio; CI, confidence interval*.

### Open-Ended Questions

In the Google form, the students were asked open-ended questions requiring them to reflect on their feelings and concerns regarding the COVID-19 pandemic. Some of the positive comments from the students were:

“*Personally, I got a lot more work done and a lot more sleep than usual.”*

“*It is great and much better than regular classes.”*

“*Everything will be OK.”*

“*I'm using my time wisely during covid-19.”*

“*The freedom of learning from home is very appealing to me.”*

Regarding the question on concerns, a majority of the concerns reported were financial in nature, such as about their ability to pay for the next semester, the possibility of increased tuition fees, and the loss of income for the provider of their family. Some students wanted the university to decrease the fee for this semester to cope with the challenges. Some of the financial concerns as expressed by the students were:

“My family business got affected by the coronavirus, and I'm having troubles in this regard.”

“I'm afraid that [the college] might increase the fees to the point where I can't afford to finish my studies.”

In addition to financial concerns, the students were also concerned about the uncertainties regarding assessments and how they would be graded.

## Discussion

College students around the world suffer from psychological morbidity, particularly depression and anxiety, due to concerns about the future and academic pressure such as managing stressful tasks and assignments and pursuit to improve their academic performance (24, 29, 30, 32, 41–44). A variety of studies have shown that college students in Saudi Arabia share the same symptoms of anxiety and stress and recorded a prevalence of depression and anxiety ranging from 14 to 50% [e.g., (25–27)]. On the other hand, Inam (45) reported around 66 and 44% level of anxiety and depression in females and males, respectively, when looking at Saudi medical school students, while Al-Gelban et al. (33) argued that 66% of the female high school students in Saudi Arabia experienced some level of anxiety. Similarly, a study by Bahhawi et al. (28) showed that students experienced some symptoms of depression (53.6%) and anxiety (65.7%) among the samples. In addition, Al Salman et al. (46) examined female secondary school students during the academic year 2018–2019 and reported around 35% level of moderate anxiety and 10% of severe anxiety.

The presence of COVID-19 is an additional factor for students to be stressed and anxious about (15). Several studies have addressed psychological well-being during the COVID-19 pandemic [e.g., (15, 47–49)] and other past epidemics [e.g., (6, 50–52)], either on students [e.g., (47)] or others [e.g., (21, 53–55)], who postulated that psychological health during pandemics must be addressed. Based on the findings of the current study, around 35% of the students experienced some level of anxiety, with 13% having severe to extreme levels. This is consistent with the research done by Alyami et al. (47), which looked at the anxiety level of the Saudi society during COVID-19 and reported 26% level of anxiety. Moreover, it is more or less similar to what was reported about students in Saudi Arabia before COVID-19 (25, 27, 28, 33). This indicates that the level of anxiety was almost consistent with pre-pandemic status. In fact, Bahhawi et al. (28) reported a higher level of anxiety. Looking outside Saudi Arabia, Cao et al. (48) found that 0.9% of college students experienced severe anxiety during COVID-19, while around 24% experienced mild to moderate levels, which presents a low level of anxiety compared to previous literature on college students in general. However, considering that Cao et al. (48) study was conducted on college students in China at the early stage of COVID-19, we can see that the lack of the full picture of this pandemic might have contributed to these results.

The argument that females are more vulnerable than men to disasters is not a new topic (56). The fact that the female students experienced higher levels of anxiety is also not surprising. Previous studies show that college female students report more stress than male students in general (28, 45, 57). In fact, comparing the two studies by Al-Gelban (25) and Al-Gelban (26) shows that using the same instrument on male and female students provided different results. While only 59.4% of the male students had one of three symptoms, 73.4% of female students had the same. We must keep in mind that the two studies are 2 years apart. Even at the post-graduate level, Almalik et al. (58) argued that female students have a significantly higher anxiety level than male students. Moreover, Huang et al. (59) argue that Chinese females experience more anxiety than males during COVID-19.

One surprising finding was the association between anxiety and the level of study; students in their fourth year were more anxious compared to students in their fifth or final year. However, one might expect that as a student progresses in his or her level of study, any consequences brought about by the pandemic would be nearly permanent and unfixable, and so fifth-year students might be more anxious than fourth-year students. Nevertheless, the fact that fifth-year students had less anxiety than their colleagues in the fourth year is also justifiable. These students were either (1) in their cooperative training program (co-op), so their graduation or academic attainment is not expected to be influenced by the virtual education decision as most of the co-op companies have arranged for an online working/training environment, or (2) in their last semester of courses; in this case, given the implementation of a special grading scheme imposed by the Ministry of Education in Saudi Arabia, a low grade point average for a student would not have an impact, regardless of the final results. Thus, these students were somehow more relaxed, at least with regard to their educational future. This explanation is supported by the reflections from some of the fourth-year respondents, such as:

“My top concern right now is whether we will be returning to the university for the first semester. It is my last semester before coop and all my courses have labs which will be near impossible to achieve in online classes.”

“I'm nervous about registering the next semester and might not have the courses that I planned to take to graduate.”

## Conclusions, Implications, and Future Research

COVID-19 has been a catastrophic experience; in the blink of an eye, this dreadful pandemic abruptly changed the way we live. As reported in the literature, pandemics are expected to have undesirable consequences not only in terms of health but also on economic, political, and educational systems (60, 61). Hence, it is imperative that the world cooperates to fight this pandemic. In that, educational institutions are advised to establish pre-outbreak policies and procedures to deal with epidemics (62).

This study is part of a more comprehensive project that aims to address the psychological well-being of university students in several parts of the world during COVID-19. The results of this study give valuable insights into the psychological status of students at a crucial time, and this, of course, has its own merit. However, it is equally crucial that future researches focus on and suggest solutions to address any effects associated with pandemics. It is important to identify appropriate strategies that could help students not only cope with adverse effects of the current pandemics but that can also enhance students' resilience to similar disasters in the future. Parents, educators, and the society as a whole should identify ways to enhance students' adaptability skills that will enable them to cope in such situations.

Moreover, future research may utilize a mixed methodology approach or large-scale comparative studies with collaborations with other countries to look at potential coping strategies that have been proven to be effective in past pandemics or during the current one (63). This might guide policy makers to develop risk management protocols as part of their policy for the future to contain future pandemics (64). Most importantly, as much as we are convinced that COVID-19 is the current enemy of humanity, we must be aware of associated impact and be able to respond effectively to all consequences.

## Limitations

This study aimed to elucidate the anxiety level of Saudi college-level students at the time of COVID-19. However, due to time constraints and to avoid a long protocol of obtaining IRB from several universities, this research only focused on one university. Although this is a small-sample study, the results can be enlightening especially since such a pandemic is a novel experience for the Saudi population, and so any data will be welcomed. The results can, hence, guide future research on COVID-19 or other epidemics.

## Data Availability Statement

The raw data supporting the conclusions of this article will be made available by the authors, without undue reservation.

## Ethics Statement

The studies involving human participants were reviewed and approved by Research and Initiative Center at Prince Sultan University. Written informed consent for participation was not required for this study in accordance with the national legislation and the institutional requirements.

## Author Contributions

HK, AA-S, and KK were responsible about data collection process. Data statistics was completed mainly by KC. The introduction, discussion, and conclusion parts were completed by all authors including HK, AA-S, KC, MN, SS, KK, GB, and SH. All authors contributed to the article and approved the submitted version.

## Conflict of Interest

The authors declare that the research was conducted in the absence of any commercial or financial relationships that could be construed as a potential conflict of interest.
